# Potential Stream Density in Mid-Atlantic U.S. Watersheds

**DOI:** 10.1371/journal.pone.0074819

**Published:** 2013-08-30

**Authors:** Andrew J. Elmore, Jason P. Julian, Steven M. Guinn, Matthew C. Fitzpatrick

**Affiliations:** 1 University of Maryland Center for Environmental Science, Appalachian Laboratory, Frostburg, Maryland, United States of America; 2 Department of Geography, Texas State University, San Marcos, Texas, United States of America; Pacific Northwest National Laboratory, United States of America

## Abstract

Stream network density exerts a strong influence on ecohydrologic processes in watersheds, yet existing stream maps fail to capture most headwater streams and therefore underestimate stream density. Furthermore, discrepancies between mapped and actual stream length vary between watersheds, confounding efforts to understand the impacts of land use on stream ecosystems. Here we report on research that predicts stream presence from coupled field observations of headwater stream channels and terrain variables that were calculated both locally and as an average across the watershed upstream of any location on the landscape. Our approach used maximum entropy modeling (MaxEnt), a robust method commonly implemented to model species distributions that requires information only on the presence of the entity of interest. In validation, the method correctly predicts the presence of 86% of all 10-m stream segments and errors are low (<1%) for catchments larger than 10 ha. We apply this model to the entire Potomac River watershed (37,800 km^2^) and several adjacent watersheds to map stream density and compare our results with the National Hydrography Dataset (NHD). We find that NHD underestimates stream density by up to 250%, with errors being greatest in the densely urbanized cities of Washington, DC and Baltimore, MD and in regions where the NHD has never been updated from its original, coarse-grain mapping. This work is the most ambitious attempt yet to map stream networks over a large region and will have lasting implications for modeling and conservation efforts.

## Introduction

Stream network density and ecohydrologic processes are closely linked through geologic and land use characteristics in watersheds [1]. Over land and through soil and bedrock substrate, water moves slowly and is subject to chemical transformations unique to conditions of continuous contact with geologic materials. In contrast, once water enters stream channels it is rapidly transported out of watersheds, reducing the amount of time for biological uptake and stream nutrient processing. Therefore, stream network density dictates both the relative importance of terrestrial and aquatic influences on stream chemistry and the residence time of water in watersheds. Models and empirical studies of watershed processes are highly sensitive to estimates of stream network density [1,2,3].

The management implications of knowing stream location and network structure cannot be overstated. At all levels of social organization, the location of streams, be they permanently or ephemerally flowing, influences land use decisions through the impact of surface water on the cost of developing land and on forest and agricultural productivity. The relationship between stream presence and land use has become more formalized over time through legislation and management of riparian buffers and wetlands [2,4]. Interpretation of such legislation has reached the United States Supreme Court, where Chief Justice Roberts stated that “where a tributary ends [the confluence] is clear; but where it begins is a problem” [5]. Such arguments and related enforcement of legislation regarding development that disturbs streams, clearly requires maps of stream channel networks with detail that matches societal understanding of what constitutes ”waters of the United States” [6]. Such maps do not currently exist for large areas, severely limiting our ability to understand, protect, and restore the ecological functioning of these natural assets.

The number and length of headwater streams in any landscape is a primary determinant of stream network density and the overall quality of any stream map is determined by its ability to resolve these small streams [7]. Many headwater streams are not included in existing hydrographical maps [8], such as the U.S. National Hydrography Dataset (NHD; [9,10]), either because they were buried during the course of urban development [11] or because their source areas were smaller than the minimum mapping size at the time of map generation. These “missing streams” severely limit the effective analysis of potential stream density based on the NHD, constituting a major problem for efforts to form regulatory policy intended to protect streams. Methods for re-mapping headwater streams and improving on the NHD include complex geospatial techniques [12,13,14,15,16], often requiring high-resolution topographic data, and detailed field and remote sensing mapping efforts [17,18,19]. Such techniques are challenging to implement over large areas [20,21], but have positioned the scientific community to make significant advances in this area. The final purpose of any stream map should influence the selection of a stream mapping technique. Perhaps the most important consideration is whether or not to assume streams are continuous, linear features [20]. For some scientific purposes, discontinuous stream segments can be accommodated. However, most practitioners require that streams be represented by continuous lines. Secondly, the union of new stream maps with older stream maps such as the NHD can provide data set continuity, but might increase the error (either commission or omission) of the resulting stream map. Finally, small-scale variability in topography can influence the identification of channel heads, the most uphill expression of streams. Such variability must either be reduced or its effect on stream map accuracy quantified if the resulting stream maps are to be used with confidence.

Recognizing the need for stream maps with spatially consistent accuracy over large geographic extents, we developed an empirically-driven workflow for mapping streams that is based solely on commonly-available geospatial data. In our approach, we do not initially assume streams are continuous features extending down gradient from channel heads. Instead, we describe a method that maps the probability of stream presence for each grid cell in a 10-m resolution digital elevation model. This technique provides flexibility in representing the topological characteristics of streams. Further, we intentionally chose a broad definition of stream [22], including all features exhibiting sorted bed load between definable channel banks. Regardless of discharge permanence, headwater channels dictate the delivery of sediments, nutrients, and pollutants to downstream waters and knowledge of their location is critical to understanding watershed processes [23], and evaluating human and ecological values of stream channels [24]. Further, although discharge permanence is often referenced in jurisdictional documents [6], it is dependent on climate variability, is temporally variable, and can be difficult to characterize over large areas.

The final product resulting from this work is a high-resolution map (derived from a 10m/pixel digital elevation model) of potential streams for the entire Potomac River watershed and all watersheds in the state of Maryland west of the Chesapeake Bay. The Potomac River was chosen because of (1) its cultural and political importance as the primary watershed supplying the city of Washington, District of Columbia; (2) its major contribution of water, sediment, and nutrients to Chesapeake Bay, the largest estuary in the U.S.; and (3) its geomorphic diversity as it flows through 5 physiographic provinces characteristic of the eastern U.S. Recent work in this geographic area has covered many aspects of headwater stream functioning and its relationship with land use change [25,26]. Indeed, the Potomac River watershed, and mid-Atlantic U.S. in general, have become a valuable outdoor experimental laboratory for addressing these issues [27,28]. We intend our results to advance ongoing research by providing a useful high-resolution stream map for this region and a new methodology for future applications in other regions. Because the NHD is used in the U.S. for nearly all work related to streams (scientific, regulatory, etc.), we compare our results with the NHD to understand its strengths and shortcomings.

## Methods

### Site description

**Figure 1:**
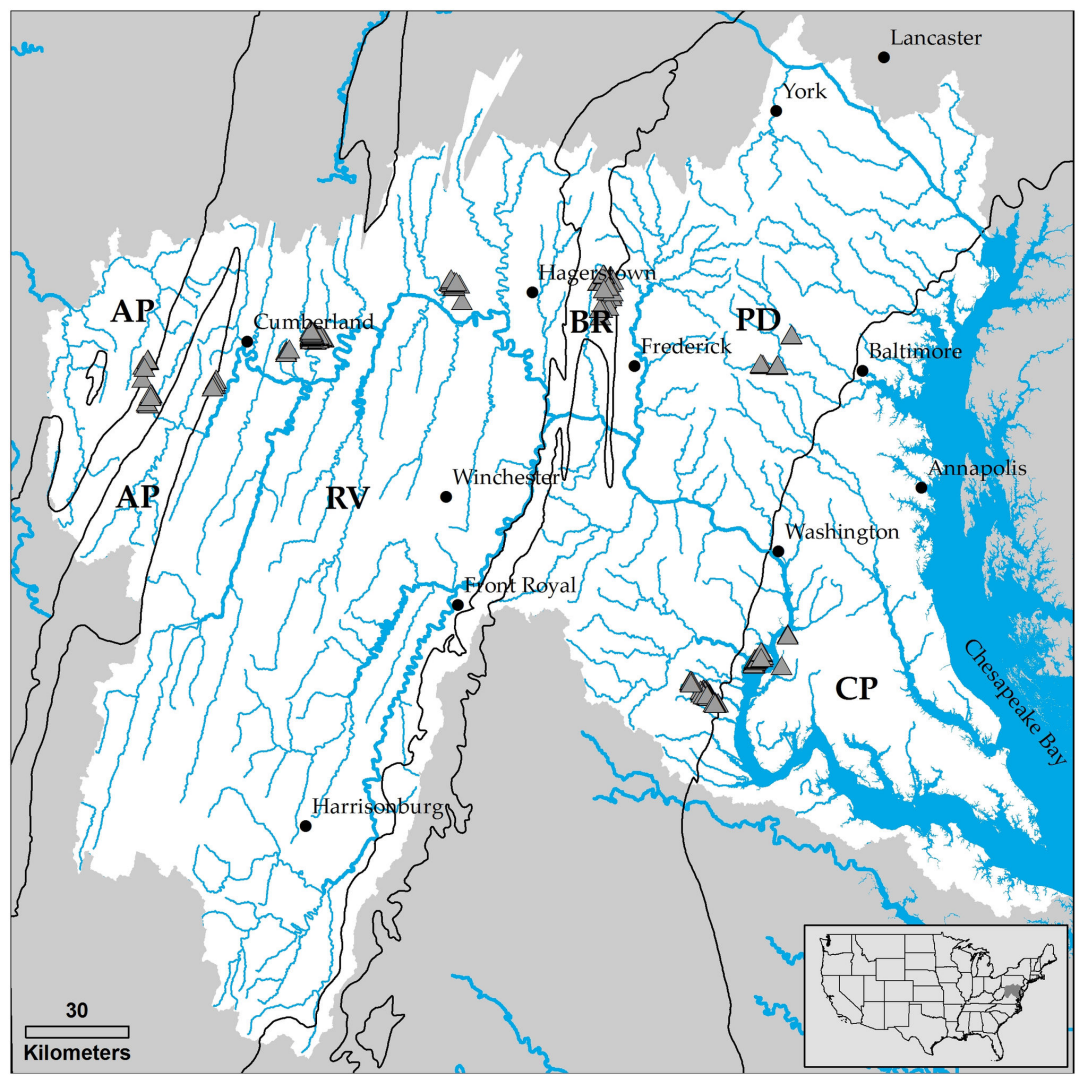
The study region covers the entire Potomac River watershed and adjacent watersheds along the Maryland–Pennsylvania boarder, and includes area in 5 distinct physiographic provinces (CP = Coastal plain; PD = Piedmont; BR = Blue ridge; RV = Ridge and valley; and AP = Appalachian plateau). Triangles denote the 253 surveyed channel heads.

The study region, covering 5.8 x 10^4^ km^2^, includes the large Potomac River watershed and 5 smaller watersheds needed to complete coverage for all of Maryland (USA) west of the Chesapeake Bay (Figure 1). The physiographic setting spans a landscape continuum from the Appalachian Mountains to the Chesapeake Bay, and includes the large metropolitan areas of Baltimore, MD and Washington, DC. As such, the study area exhibits considerable geologic and land use diversity [29]. The Potomac River serves as the major water source to the large metropolitan population of the U.S. capital and is the second largest source of freshwater input to the Chesapeake Bay [30].

The study region includes 5 physiographic provinces (Figure 1), each with a distinctive geology and land cover influencing stream network density [31]. The mostly forested Appalachian Plateau (AP) is underlain by sandstone, shale, conglomerate, and coal. Extreme folding and faulting in the adjacent Ridge and Valley (RV) has created roughly parallel ridges and valleys composed of a variety of geologic materials, including limestone, shale, siltstone, sandstone, chert, and mudstone. Present land cover here is a mix of forest (mostly oak-hickory) and agriculture. The heavily forested Blue Ridge (BR) is characterized by steep, rugged terrain over mostly metamorphic rocks. Numerous springs exist throughout this province, particularly along fractures and at the boundaries between lithologic units. The Piedmont (PD) is characterized by irregular plains and moderate relief with thick, clay-rich, soils underlain by deeply weathered bedrock. The Coastal Plain (CP), which is adjacent to the Chesapeake Bay, is relatively flat with the exception of a gentle slope extending from its contact with the Piedmont to the Bay, including several terraces formed by former oceanic shorelines. The CP is underlain by a thick layer of sediments (gravel, sand, silt, and clay) that tend to be poorly drained, particularly in the lowlands of the outer CP. The inner CP constitutes the more upland areas and consists of fluvial-deltaic sediments (gravel, sand, clay) formed as large rivers from the west eroded to the sea. Lowland areas contain swamps and marshes, while mixed forests dominate upland areas. Land cover in the Piedmont and Coastal Plain is mixed, with much larger urban areas compared to the other three provinces.

### Landscape variables

We derived terrain variables (accumulation area, topographic slope and curvature) from 10-m National Elevation Data (NED [32]) that were expected to provide spatial information useful for predicting the location of stream channels [20]. To these, we added one soil property (silt + clay percentage [33]) expected to relate to soil erodibility and permeability. Terrain processing included the following steps [34,35,36]: (1) development of a hydrologically corrected digital elevation model (10-m resolution) (2), flow direction modeling (3), flow direction enforcement to NHD flowlines, e.g., [37], and (4) flow accumulation calculation for each 10-m pixel. For each landscape variable, we calculated both a local value and an accumulated value, which was the mean of all pixels contributing flow to each location. Each landscape variable was therefore represented by a continuous raster, covering the entire study region at 10-m spatial resolution.

### Field surveys of stream channels

Field surveying of channel heads and stream presence data, including quality assurance, are detailed in Julian et al. (2012). Briefly, we collected the geographic position of 253 channel heads located on public land across the five physiographic provinces of the mid-Atlantic U.S. and identified the closest pixel to each field surveyed location that, based on its flow accumulation area, could realistically be considered the intended channel head location. We studied the location of channel heads on plots of landscape variables (local slope, plan and profile curvature, etc.) against flow accumulation area, and presented our observations in a previous publication [9]. From the resulting database of channel head locations we identified stream presence by labeling all pixels down flow direction from channel heads as streams. In this way, small watershed maps were generated for all areas where field data were available. Mapped watersheds, which in some cases contained several small tributaries, varied from 1.2 to 814.1 ha, contained between 1 and 46 channel heads each, and cumulatively covered 62.6 km^2^. Because we noted only the presence of stream channels and did not record absence, we used a stream prediction algorithm (see next section) that requires presence-only data and uses in the place of absences ‘background’ points selected at random from the landscape. In this application the total number of stream presence observations was 10,565. No specific permits were required for the described field surveys. No samples were collected (e.g., invertebrates, plant species, corals, or non-living materials.)

### Predicting the location of stream channels

The capability to predict the location of channel heads from topographic variables leads directly to stream maps if and only if streams are assumed to be continuous linear features [20] (i.e., if channel head locations are known or predicted, streams can be represented as continuous lines flowing down gradient). On the other hand, there are many methods for predicting the presence or absence of geomorphic or biologic features on the landscape that do not require spatial continuity [38,39], but can be made continuous after the fact if so desired. Such methods are generally lumped into the category of species distribution models. Previous work compared these two approaches to mapping streams [20]. We seek to advance the science by selecting a relatively novel species distribution model for predicting the probability of stream presence.

We related the landscape variables to stream channel occurrence data using MaxEnt 3.3.3e [40]. MaxEnt is an implementation of a statistical approach called maximum entropy that characterizes probability distributions from incomplete information. In this application, the incomplete information is a geographic sample of the topographic and soil variables associated with the presence of stream channels on the landscape. In the context of modeling stream networks using maximum entropy, the assumptions are that (1) stream occurrence data represent an incomplete sample of an empirical probability distribution describing the probability of stream presence in a given environment, that (2) this unknown distribution can be most appropriately estimated as the distribution with maximum entropy (i.e. the probability distribution that is most uniform) subject to constraints imposed by terrain and soil variables, and that (3) this distribution of maximum entropy approximates the potential occurrence of stream channels on the landscape. MaxEnt minimizes the relative entropy between the probability density of environments where stream channels are known to be present and the probability density of environments estimated from the landscape as a whole from the randomly generated background data (for more details, see 40,41). MaxEnt has most commonly been used to model and map species distributions. While other species distribution modeling techniques have been used to model the occurrence of geomorphic features [42,43,44], to our knowledge this is the first MaxEnt application to do so.

The field survey data (10,565 individual 10-m resolution pixels with stream presence observations) and 50,000 background points were split randomly (70%-30%) into model training data and testing data, respectively. To fit models, we used 10-fold cross-validation and the default value for the convergence threshold (10^-5^) suggested previously [40]. The maximum number of iterations was set to 5000. Determination of regularization values, which address problems of over-fitting and selection of ‘features’ (terrain and soil variables and/or functions derived from combinations of such variables) were performed automatically by the program per the default rules. The MaxEnt procedure resulted in maps of the predicted probability of stream presence (as estimated using logistic output from MaxEnt [41,45]), information on the relative importance of the landscape variables for predicting streams, and Area Under the Curve (AUC; [46]) of the receiver-operating characteristic (ROC) plot for evaluating predictive performance using training and testing survey data. To calculate and interpret of the importance of landscape variables for predicting streams, we ran a second MaxEnt model that used survey data from each physiographic province separately. For this second case, we used 10,000 background values for each physiographic province, but kept all other parameters the same as above.

Stream probability did not always increase continuously when moving downstream along stream channels because curvature and slope values along flowlines with small contributing areas can vary substantially across short distances (due to both DEM errors and actual spatial variability). We considered it important to apply an *a priori* constraint that stream channels be continuous landscape features. This, of course, is not always the case as channels do occasionally become discontinuous along flow lines. However, stream maps with discontinuous channels are considered undesirable by practitioners and are only used in special cases. We addressed the occurrence of discontinuous stream channels in a two-step process. First, we implemented a filter along flow lines for all MaxEnt probabilities greater than 0.01 that replaced the probability of stream presence at each location with the mean probability of the 3 pixels (one upstream and one downstream) centered on that location. This procedure had the effect of smoothing the probability of stream presence along each flow line, primarily reducing the occurrence of isolated pixels with a high stream probability. Second, after applying a threshold (the calculation of which is described below) to the stream probability maps, we connected all discontinuous segments of steam presence along flow lines, effectively delineating streams beginning at their most probable upstream prediction.

To map stream locations, the continuous output (0-1) from MaxEnt had to be converted to predicted presence (1) or absence (0) and a probability threshold of 0.5 does not necessarily result in the best fit to the data [47]. Beyond providing a measure of model performance (AUC), the ROC plot can also inform the selection of thresholds. As typically implemented, the ROC is a plot of the true-positive prediction rate (sensitivity; equivalent to 1 – false negative rate) versus the false-positive prediction rate (1 – specificity) across all possible threshold values [46]. A threshold that maximizes true positive predictions while minimizing false positive predictions is desirable and can easily be identified from the ROC plot as the point closest (Euclidian distance) to a true positive rate of 1 and a false positive rate of 0. However, this approach assumes that the costs of false positive and false negative errors are equivalent [48,49]. Our field survey exhibited vastly differing areas of stream and non-stream (~ a 1:60 ratio) with many of the non-stream locations receiving very low MaxEnt probabilities (i.e., < 0.01). Using a traditional ROC plot, true positive and false positive predictions would be weighted 1:60, but we desired these to be weighted more equally. Therefore, we used a modified ROC plot to determine the threshold that maximized true positive accuracy while limiting false positive errors. This was accomplished by normalizing the number of false positive predictions by the number of stream survey points (rather than the number of *non-stream* survey points as is done in a traditional ROC plot), effectively limiting the area that could be predicted as streams to the area of streams in the field survey [50]. The result was a higher threshold than would be obtained from a traditional ROC plot and therefore a more conservative estimate of stream presence across the landscape. For this analysis we used the field survey data in each physiographic province separately as a check on the robustness of the calculated thresholds. Following Maxent modeling, threshold selection, and flow-line filtering and cleaning to connect discontinuous stream segments, predictive performance was assessed using the field survey data as 1-omission error (false negative) rates.

### Generating summary performance statistics

We calculated true and false prediction rates (number of true and false predictions divided by the number of stream survey locations) and false negative rates (number of false predictions divided by stream survey locations) for the resulting stream map. To evaluate how each step in the workflow influenced the result, we also calculated these statistics for (1) raw MaxEnt results, (2) MaxEnt results after smoothing and connecting discontinuous stream segments, and (3) after merging with NHD maps of streams. Finally, we calculated a second stream map based on a critical threshold flow accumulation area at the channel head (Ac). For this map, we referenced our field survey of streams and calculated the average Ac for each physiographic province. We then adopted this value as the “Tuned Ac” and mapped streams as all pixels with a flow accumulation area greater than the Tuned Ac for each physiographic province. We then generated summary tables for each physiographic province detailing the true and false prediction rates for each map (including NHD alone) for both the entire area and for non-NHD streams.

We calculated stream density for each data set at the Hydrologic Unit Code (HUC) 12 level by dividing the stream length (km) by the watershed area (km^2^). The increase in stream density attained by the modeled streams over the NHD was calculated as a percentage, both across entire watersheds and as a function of flow accumulation area. We also calculated the total number of channel heads in each watershed. We compared, through linear regression, the percent increase in stream density and channel head density against average land cover values (derived from the 2006 National Land Cover Dataset [51,52]) for each watershed.

## Results

**Figure 2:**
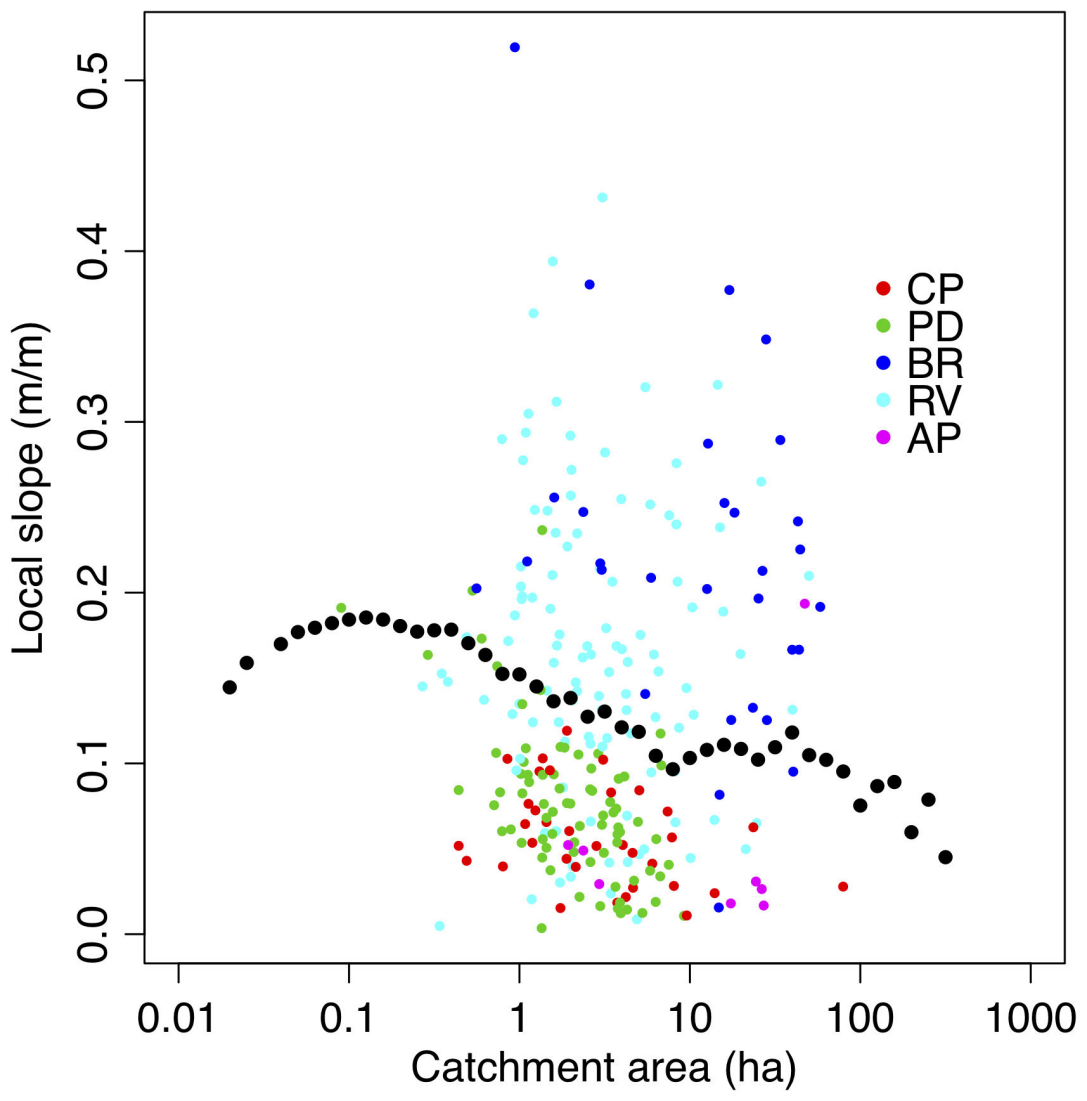
Local slope vs. Log_10_(catchment area) for bins of increasing catchment area (black dots) across the entire survey region and for the locations of channel heads (colored dots). Characteristics of this plot have been discussed previously in the literature [21,53,64], and are covered briefly in the text.

Surveyed channel heads were located at catchment areas between approximately 1 and 100ha, which was a similar range to what has been found in other environments [53]. In the Appalachian Plateau and Piedmont the local slope and catchment area at the channel head locations were negatively correlated [9], but generally the surveyed channel heads exhibited a large range and variability in the catchment area at the channel head (Figure 2). This variability could be due to any number of factors (e.g., geologic controls on groundwater-surface water interactions, climate, historic land-use), and precluded attempts to directly predict the location of channel heads using available landscape data. Previous successful attempts in this pursuit might be attributed to less spatially variable geology [21,54]. This observation justified our approach to stream mapping, that (as described above) utilized all the information extracted from the location of streams to build a predictive model for stream presence pixel-by-pixel via MaxEnt modeling [20].

Analysis of variable contributions within MaxEnt indicated that flow accumulation area had by far the largest relative contribution (96.8% overall) to the model of stream presence. Of distant secondary importance were average plan curvature (1.5%) and average slope (0.8%). Variables averaged over the entire catchment always had greater contribution to the model than local variables, the latter of which in all cases did not contribute to the predictions of stream presence. MaxEnt performance was very stable, with the AUC equaling 0.934 for both training and test data separately. When a separate MaxEnt model was run for each physiographic province, the rank order of variable contribution was similar to the lumped model. However, the average plan curvature was more important in the Coastal Plain and the Piedmont than elsewhere in the study region (Table 1). Notably, only in the Coastal Plain was the contribution of source area below 85%. Critical thresholds in the MaxEnt probability distribution that maximized the true positive rate while minimizing the false positive rate were similar for each province (0.44 (AP), 0.48 (RV), 0.50 (BR), 0.46 (PD), and 0.48 (CP)). These thresholds were used in all subsequent accuracy assessments (e.g., Tables 2 and 3).

**Table 1:** Permutation importance from MaxEnt for each parameter across the physiographic regions.

**Parameter**	**CP (952)**	**PD (2206)**	**BR (2870)**	**RV (4176)**	**AP (359)**
Source Area	82.1	98	97.8	96.3	92.9
Average Plan Curvature	11.3	0.5	0.3	2.1	3.2
Average Slope	5.6	0.7	1.2	0.3	2.5
Average Profile Curvature	0.5	0.6	0.5	1	1
Local Slope	0.2	0	0	0.2	0.2
Percent Clay + Silt	0.1	0.1	0.1	0.2	0.1
Local Profile Curvature	0	0	0	0	0
Local Plan Curvature	0	0	0.1	0	0

Stream observations (n) used to fit the model in each physiographic province are given in parentheses.

**Table 2:** False Prediction Rates (FPR) and True Prediction Rates (TPR) for stream maps generated for a threshold flow accumulation area (Tuned Ac), the NHD, and MaxEnt-modeled streams at three stages in the workflow.

**Province**	**Tuned Ac**		**NHD**		**Raw MaxEnt**		**Smoothed & Connected MaxEnt**		**Merged with NHD**	
	**FPR**	**TPR**	**FPR**	**TPR**	**FPR**	**TPR**	**FPR**	**TPR**	**FPR**	**TPR**
**CP**	36	76	18	62	16	81	16	84	25	86
**PD**	28	90	2	45	9	87	9	88	10	88
**BR**	21	85	22	69	15	70	19	84	27	87
**RV**	18	81	5	48	17	83	16	84	17	84
**AP**	36	92	26	80	11	71	13	72	27	88
**ALL**	24	84	13	55	14	80	16	84	20	86

Ideal FPR and TPR are near 0 and 100, respectively

**Table 3:** False Prediction Rates (FPR) and True Prediction Rates (TPR) for non-NHD streams only.

**Province**	**Tuned Ac**		**Smoothed & Connected MaxEnt**	
	**FPR**	**TPR**	**FPR**	**TPR**
**CP**	73	71	37	64
**PD**	44	81	17	78
**BR**	60	68	42	58
**RV**	35	69	28	70
**AP**	84	68	36	41
**ALL**	50	72	29	69

**Figure 3:**
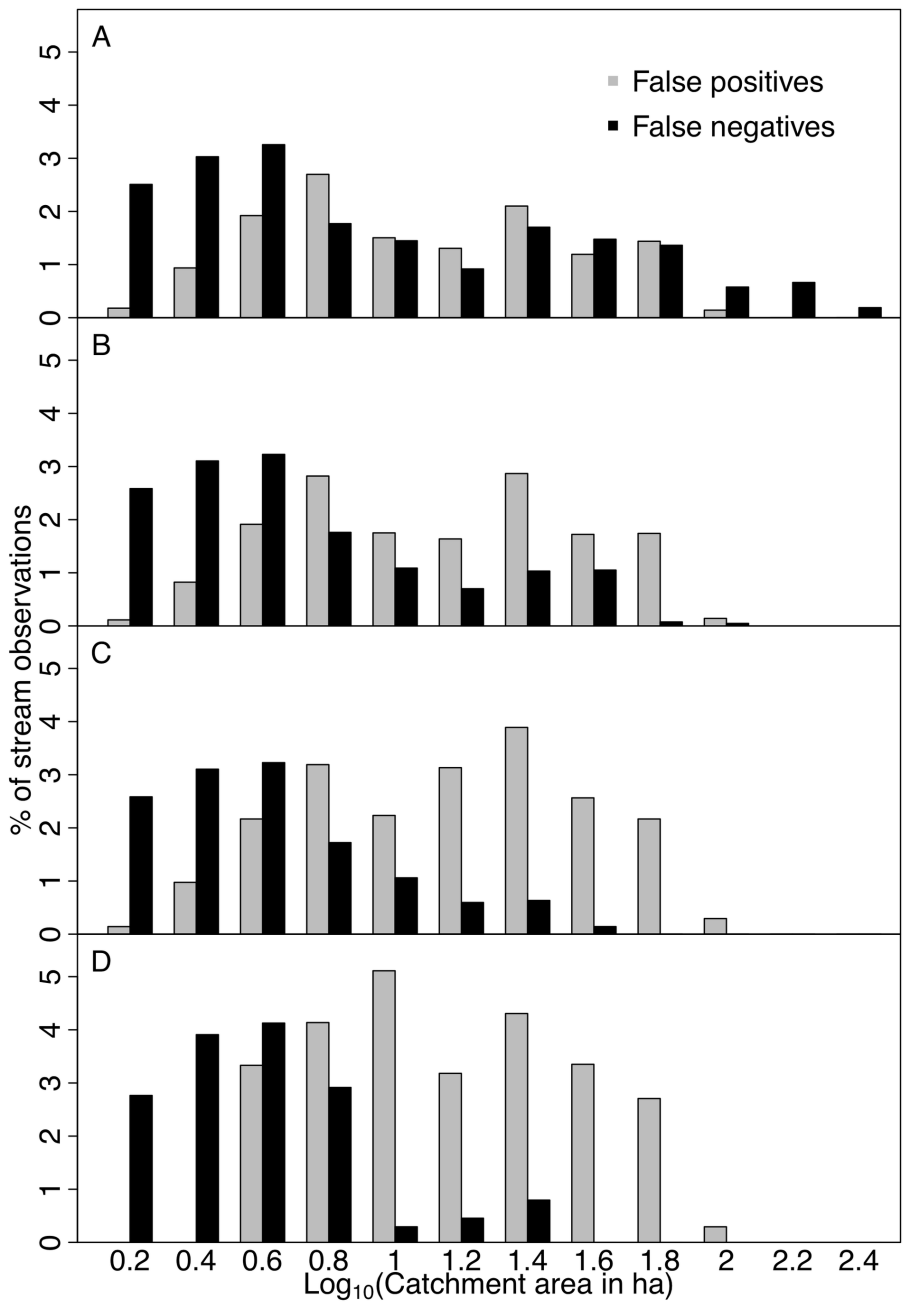
False positive and false negative predictions, expressed as a percentage of stream observations in the field survey (10,565), for four stream maps: (A) raw MaxEnt results with the province-specific thresholds applied; (B) MaxEnt results after smoothing and connecting discontinuous stream segments; (C) the results in (B) after merging with NHD maps of streams, and (D) the Tuned Ac stream map, which uses a province-specific critical catchment area to define streams. Methods for each map are described in detail in the text.

The complete stream mapping workflow delineated 1.48 x 10^5^ km of stream length across the study region, which included 1.19 x 10^5^ km of streams in the Potomac River watershed and 5.38 x 10^4^ km in the state of Maryland west of the Chesapeake Bay. Prior to connecting discontinuous stream segments, false positive and negative predictions were balanced across a large range in catchment area (Figure 3A). True prediction rates (a measure of accuracy; Table 2) were in the range of 70-87%, indicating that MaxEnt miss-classified a quarter of all surveyed streams. After connecting discontinuous stream segments, the percentage of field-surveyed streams correctly predicted was 84% (of the 10,565 stream survey pixels) with a false prediction rate of 16%. Accuracy varied slightly by physiographic province (Table 2) with the lowest accuracy achieved in the Appalachian Plateau (72%). Most omission errors occurred in small streams with a catchment area smaller than 10 ha (Figure 2B). For these small streams, errors of commission and omission (false positives and false negatives, respectively) were roughly in balance at a catchment area of 3-4 ha (Log_10_C_A_ = 0.6; where C_A_ = catchment area in ha), which is similar to what it was before connecting discontinuous stream segments. At any given catchment size (Log_10_C_A_ bin size = 0.2) errors of either type were generally below 3%. False prediction rates increased after discontinuous stream segments were connected, but only moderately so considering true prediction rates increased as well. For example, for the Blue Ridge true prediction rates increased from 70 to 84% with the connection of discontinuous segments. At the same time, false prediction rates increased from 15 to only 19%, suggesting that connecting streams corrected the classification more often than not. NHD alone exhibited a false positive rate between 2 and 26% and an overall true positive rate between 45 and 80% (Table 2). Despite this low performance, as a final step, we merged the MaxEnt stream map with the NHD. Therefore, the effect of joining the two maps (MaxEnt and NHD) was to increase the false positive rate (substantially in some provinces), but to increase the true positive rate as well (Table 2, Figure 3).

We compared the performance of the connected MaxEnt stream map with a map generated using a critical flow accumulation area (Ac), tuned to each physiographic province (termed the “Tuned Ac” map). The Tuned Ac map exhibited a higher false prediction rate (between 18 and 36%) and a similar true prediction rate (between 81 and 90%) compared with the MaxEnt map. Because, the location of large streams is easier to predict than small streams, we removed all NHD streams from the field survey and compared the true prediction rates and false prediction rates of the remaining area between these same two maps (Table 3). The Tuned Ac map exhibited higher false prediction rates for these small, non-NHD streams (between 35 and 84%), but similar true prediction rates compared with the connected MaxEnt stream map. In summary, the advantage of the MaxEnt map was lower false prediction rates across most of the study region at the expense of only slightly lower true prediction rates. This can be seen graphically in Figure 3C and 3D, which shows increased false positive predictions over false negative predictions in the Tuned Ac map, particularly for streams in catchments larger than 10 ha (Log_10_C_A_ = 1).

**Figure 4:**
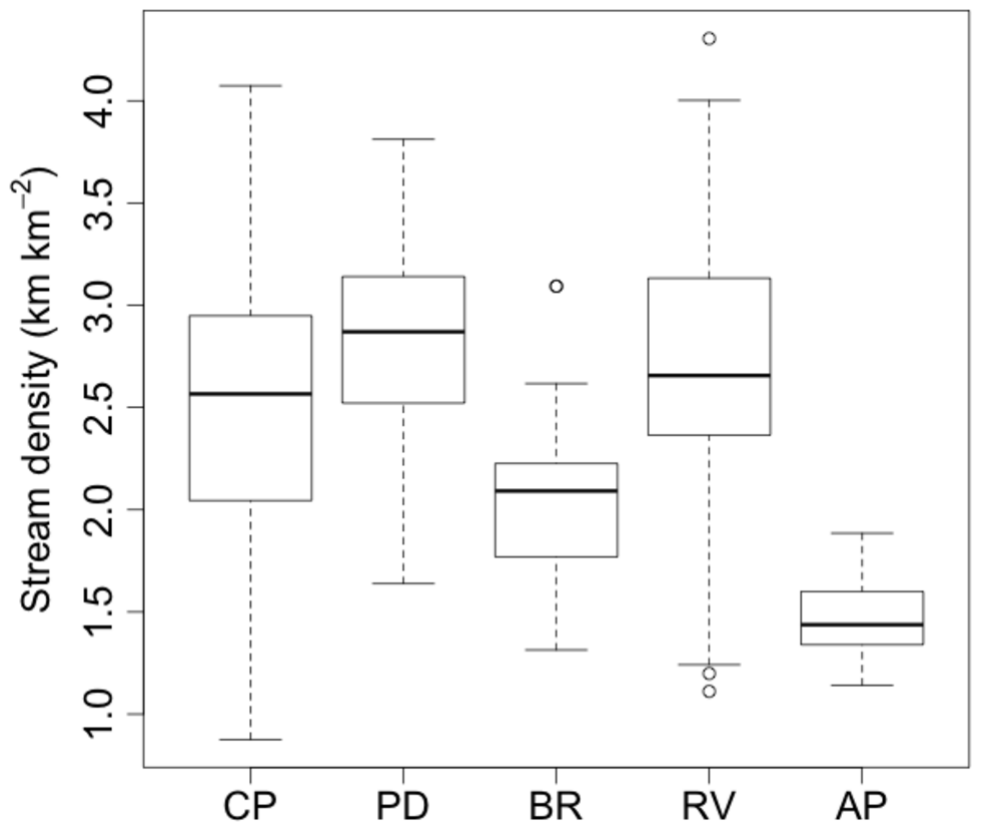
The mean and variance of stream density for HUC12 watersheds by physiographic province (CP = Coastal plain; PD = Piedmont; BR = Blue ridge; RV = Ridge and valley; and AP = Appalachian plateau).

**Figure 5:**
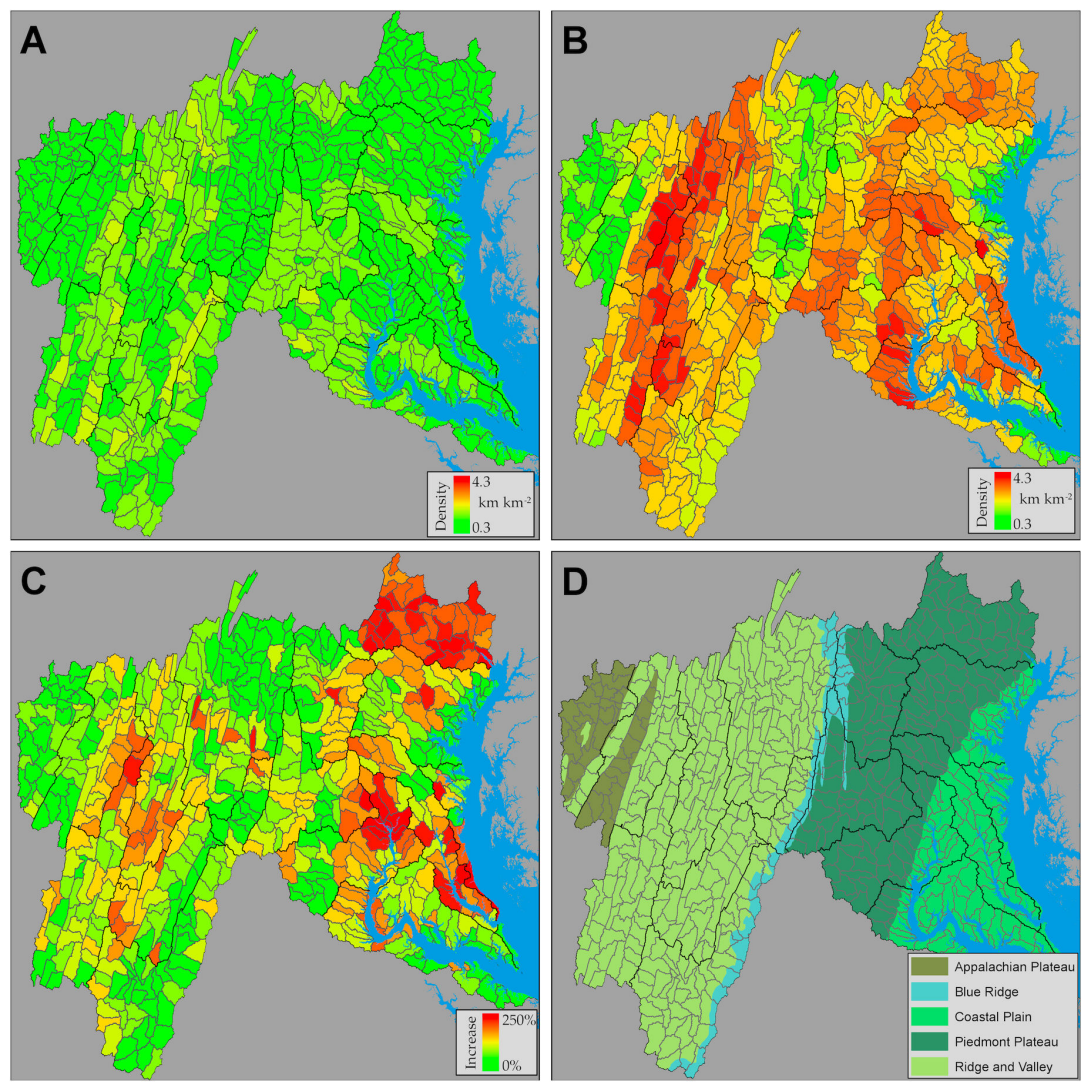
Stream density maps for HUC12 watersheds in the study region. (A) NHD stream density was more uniform and lower than (B) the stream density calculated from MaxEnt after smoothing, connecting discontinuous segments, and merging with NHD. (C) The percent change in stream density highlights the effects of urban areas and other areas with poor quality NHD stream maps. (D) Spatial variation in stream density can be explained in part by differences in geology between physiographic provinces.

**Figure 6:**
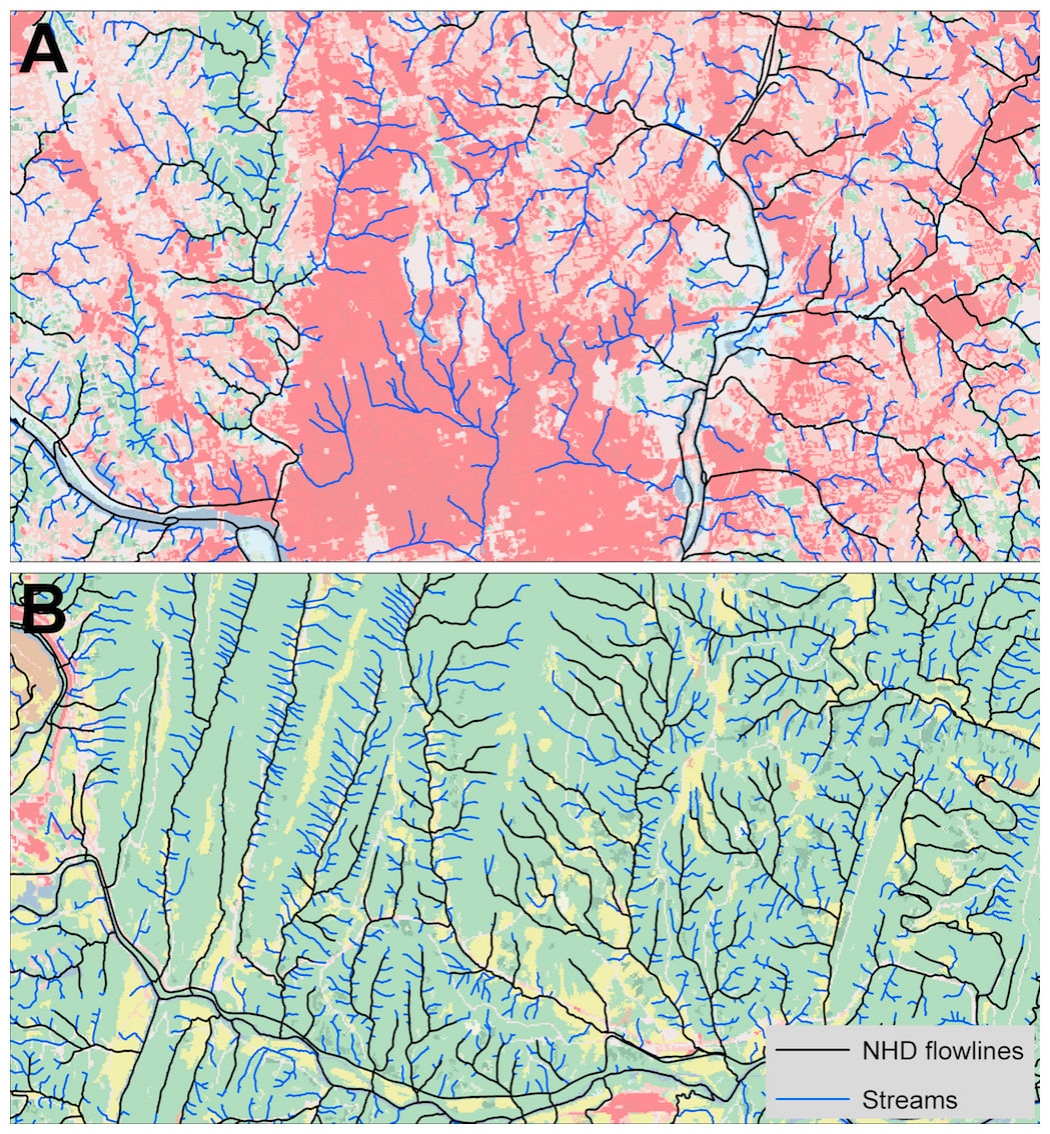
Examples showing the density and configuration of the streams mapped in this study (blue; MaxEnt streams, smoothed and connected as in Figure 3B) compared with the NHD streams (black) for (A) an urban area and (B) a rural area. The background image shows the 2006 NLCD land cover using the standard color scheme: urban (shades of red), forest (green), agriculture (yellow), and water (blue).

Across physiographic provinces, the heavily dissected Piedmont and Ridge and Valley provinces, which are covered by highly weathered soils, exhibited the highest stream density (Figure 4). Slightly lower stream densities were found in the groundwater-dominated Coastal Plain, and much lower stream densities were found in the more bedrock dominated Appalachian Plateau and Blue Ridge. Maps of stream density at the HUC12 scale (using the complete workflow, including merging with NHD) exhibited substantial within province variation (Figure 5). Mapped streams exhibited higher stream density relative to the NHD streams (Figure 5B and 5C). Streams added to the NHD were generally smaller first- and second-order tributaries that flow into larger streams (Figure 6). Elsewhere, new streams were mapped as extensions of existing NHD streams. These two types of stream additions to the NHD resulted in stream density increases of up to 250% over the NHD. Increases were large in the metropolitan areas of DC and Baltimore, where streams have been buried beneath urban land cover since before the original NHD mapping. The increase in stream density was also large in the northeast portion of the study area (i.e. Deer Creek – Susquehanna watershed).

**Figure 7:**
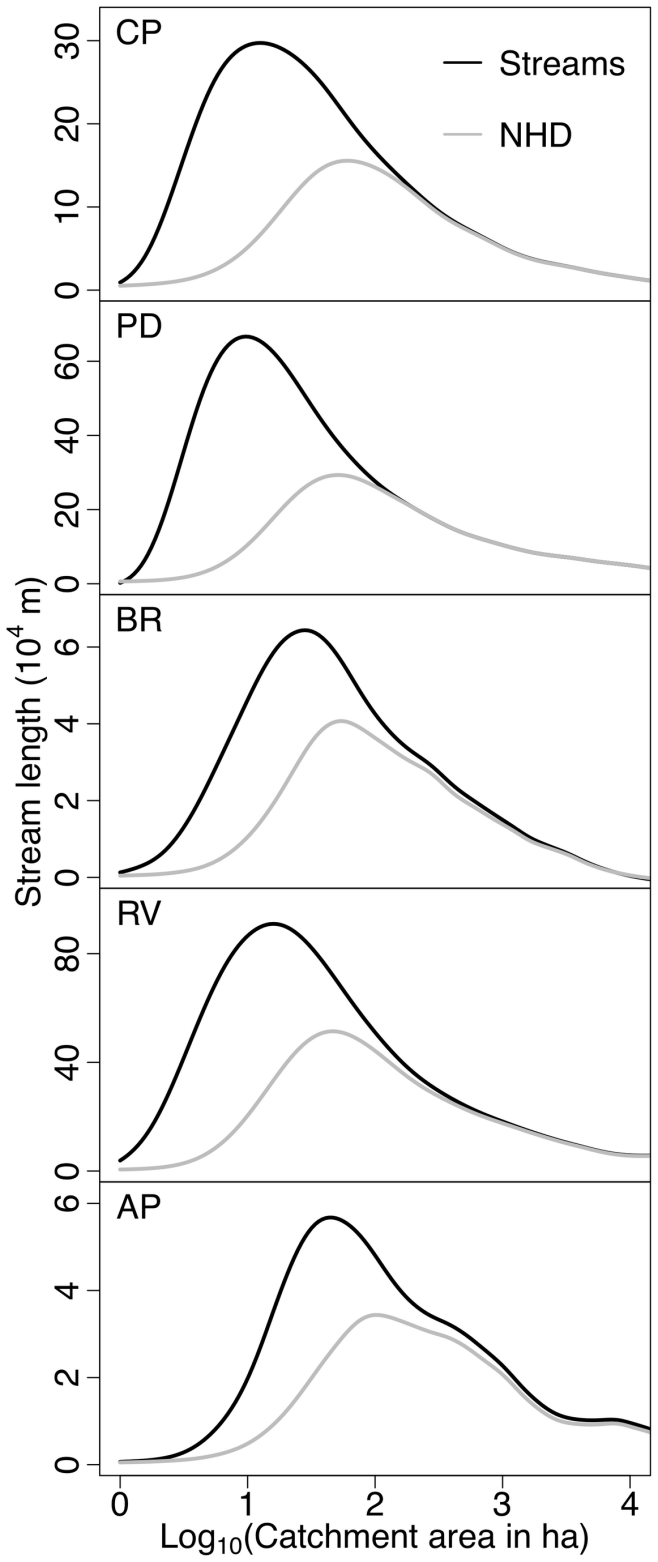
Stream length as a function of the log catchment area for each physiographic province (CP = Coastal plain; PD = Piedmont; BR = Blue ridge; RV = Ridge and valley; and AP = Appalachian plateau).

**Figure 8:**
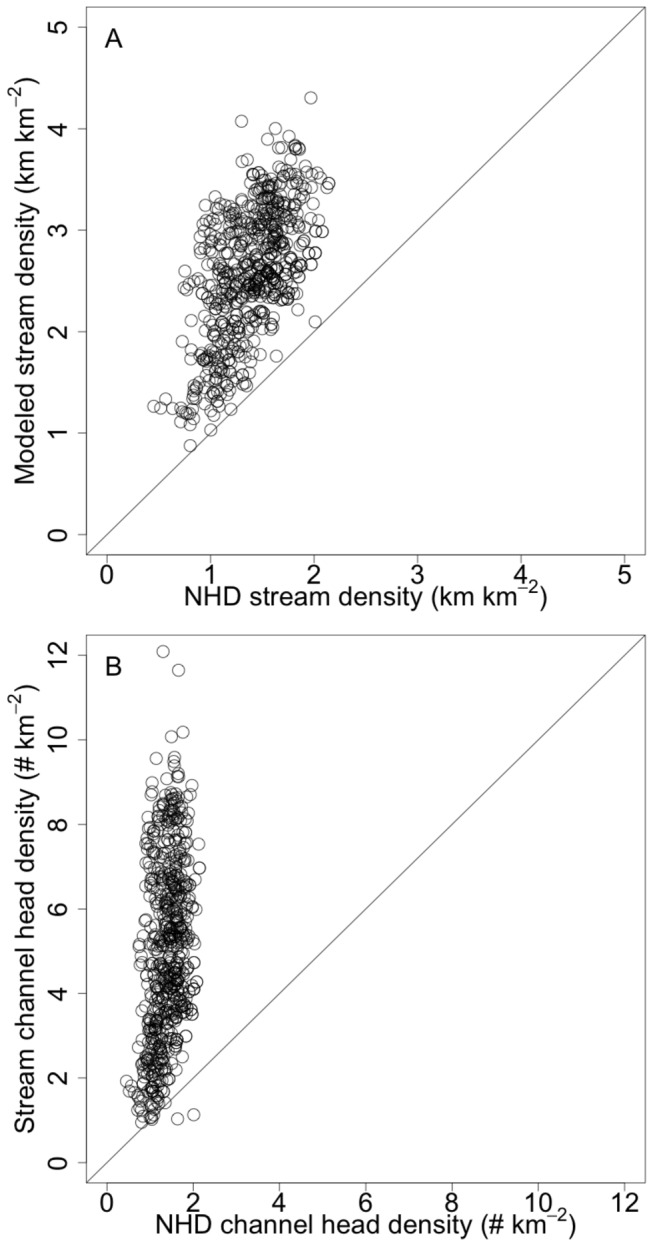
Modeled vs. NHD stream density metrics for each HUC12 watershed in the study area. In (A) modeled stream density is compared to NHD stream density, and in (B) modeled channel head density is compared to NHD channel head density. Although there is a strong correlation, modeled streams exhibit many more small streams per unit area, each with its own channel head.

Relative to the NHD, mapped streams exhibited a distribution that was weighted towards smaller streams (Figure 7). This weighting varied predictably by physiographic province with more small steams being added to the stream map in the Piedmont, Ridge and Valley, and Coastal Plain than in the other two provinces. At the HUC12 level, potential stream density correlated with NHD stream density, but was strongly offset from the 1:1 line (R^2^ = 0.36; *P* < 0.001; Figure 8A). The density of channel heads was also correlated between NHD and modeled streams, but with the modeled streams exhibiting approximately 10 channel heads for every one in the NHD (R^2^ = 0.11; *P* < 0.001; Figure 8B). Although the cities of Baltimore and DC exhibited low stream density in the NHD map, in general, the percentage increase in stream density across all HUC12s did not correlate with urban area, forest area, or any other landscape variables used in the analysis.

## Discussion

The effect of watershed stream density dominates over many watershed processes, including biogeochemical and biologic responses to land use and climate change, and atmospheric deposition of contaminates. The number and length of the smallest streams in a watershed, for example, determines the proximity of any land use activity to a stream. When all channels are recognized, the placement of roads and urban structures without crossing or covering streams becomes problematic. Small streams are less expensive to fill and build over, leading to stream burial rates that increase with decreasing stream size [11]. Overall, stream density is a reasonable indicator of the vulnerability of watersheds to land use change. Evidence that the Coastal Plain of Maryland, which exhibits lower stream density than the adjacent Piedmont (Table 1), is more resilient to the effects of urbanization and associated changes in stream biology is consistent with this idea [55]. At finer spatial scales, work has shown measurable impacts from road crossings and riparian buffer infringements across a large range of road and stream sizes [56,57]. Such work supports the notion that with better information on the locations of stream channels, land can be strategically developed to limit impacts to aquatic ecosystems.

The stream map produced here is a representation of the potential stream density of the mid-Atlantic region. This density would be achievable only if the entire study region had the same current and historic land use as the forested watersheds used to train our model. However, land use has undoubtedly influenced stream density away from this potential density, increasing stream length in some locations and decreasing it in others. In largely forested watersheds, streams lengthen wherever low-density development (country roads, exurban development, etc.) increases runoff and concentrates flow through culverts [58,59]. In more heavily developed areas, stormwater systems divert flow away from streams and manufactured drainage structures, often replacing the potential stream network [11]. Finally, in densely urbanized areas entire rivers are directed underground. Because our map represents the potential stream density, it is ideally suited to study the extent and magnitude of these processes at landscape scales.

The new potential stream maps faithfully represent spatial variation in stream density among physiographic regions, therefore reflecting variation in stream habitat area and the landscape connectivity of streams. Spatial variation in stream density is influenced by topography, which in turn is set by the geology of an area. The largest modes of spatial variation in stream density were found at the scale of physiographic provinces (Figure 4). Within-province variation was substantial in some provinces, such as the Ridge and Valley and Coastal Plain. Because we used entirely forested watersheds to train our predictive model, this within province variation is not due to land use at this scale. Instead, variation in topographic variability and the underlying geology likely cause spatial variation in stream density. In the Ridge and Valley, for example, variable geology (including karst) influences the relative contributions surface and groundwater have on stream channel formation [9].

A primary component to our stream mapping procedure were predictions of stream presence from MaxEnt, an approach widely used in ecology and biogeography to predict species distributions [60,61]. While numerous algorithms exist for such predictive spatial mapping, most of which have seen limited use in geomorphology [42,43,44], Maxent has the advantage of using data only on the presence of the feature of interest, while selecting random background data as part of model fitting and evaluation. MaxEnt also has been found to be among the top performing methods in terms of predictive accuracy [39,62]. Some of the same landscape variables used here (e.g., landscape curvature and slope, soil characteristics) have been used to predict lake depth [63], stream distributions [20], and a suite of geomorphic features [42]. Not surprisingly, we found source area to have the greatest relative contribution due to its strong control on hydrologic discharge. Of secondary importance were average slope and plan curvature, supporting the use of simple slope-area relationships to predict the source area at channel initialization [54]. The average plan curvature and average slope were most important in the Coastal plain and Appalachian plateau (Table 1). Variables other than source area likely had the greatest impact on the resulting maps in areas where source area was less than 10ha. For catchments greater than 10ha in size, omission errors approached zero, indicating that source area had reached a critical size and was dominating model performance. A comparison among different methods and a closer look at their structure and capabilities in predictive geomorphological mapping is warranted.

Ideal stream maps will exhibit high true prediction rates (e.g., near 100%) and low false prediction rates (e.g., near 0%). The Tuned Ac and NHD maps both exhibited large variation in accuracy between physiographic provinces (Table 2). Further, the Tuned Ac map tended to over-predict stream presence, with larger false prediction rates than other maps. The NHD exhibited low overall accuracy, but unexpectedly also exhibited large false prediction rates in the Coastal Plain, Blue Ridge and Appalachian Plateau physiographic provinces. MaxEnt-derived stream maps exhibited true positive rates comparable to the Tuned Ac map, but with a lower false positive rate. Further, we found that by connecting discontinuous stream segments it was possible to increase true prediction rates, largely without increasing false predictions. We were pleased with this result given the importance maintaining low false predictions while also maintaining a conservative estimate for total stream length. When we merged the connected MaxEnt stream map with the NHD, the false predictions inherent to the NHD in some provinces were added to the model-based map (which was undesirable), but true prediction rates increased for some of the least accurate physiographic provinces (e.g., the Appalachian plateau.) Overall, we would be working with a better map if we didn’t merge the MaxEnt results with the NHD, but due to the acceptance the NHD map has gained in regulatory policy and practice it continues to seem prudent to include it in any mapping effort that would be used outside of basic science inquiry.

We also compared false and true positive prediction rates for only those stream segments that were not also in the NHD (Table 3). Non-NHD streams are typically smaller and include most of the first-order channels in the field survey (Figure 6), which tests the capabilities of the models and reveals inherent weaknesses. Consequently, the false and true prediction rates were far from ideal for both the Tuned Ac and MaxEnt (connected) models. The Tuned Ac map exhibited a higher true prediction rate than the MaxEnt map, but at the expense of higher false predictions. One might argue in fact that the Tuned Ac false prediction rate for non-NHD stream segments was so high (reaching 84% in the Appalachian Plateau) as to be unusable for any purpose. Although the false prediction rates of the MaxEnt map were more manageable (ranging from 17 to 42%), true prediction rates for non-NHD streams were low for several provinces, thus leaving room for improvement. One improvement we see as necessary is better non-topographic environmental data. Our maps of soil texture and physiographic province, for example, are generated at a lower mapping resolution than any of the other variables. Further advances in stream map accuracy might also be possible with the inclusion of more field survey data; mapping of channel heads is a time and labor consuming process that might be best attempted using citizen scientists or other forms of crowd sourced information.

High-quality data on the landscape distribution of natural resources is a prerequisite to sustainable development. In the U.S., land use decisions are made with regard to stream presence using the NHD, which is publically available to all levels of government and society. Unfortunately, the NHD is inconsistent in its representation of streams, and in our study region, under-represents total stream density by as much as 250%. This underrepresentation is sometimes due to urbanization that occurred prior to the original stream maps being generated. However, even within forested watersheds there is wide variability in the accuracy of the NHD. For example, in the northeastern corner of the study region the NHD exhibits a low stream density, apparently a result of the original 100-k scale mapping, which has never been updated. A detailed analysis of the impact this inaccuracy has had on land use decisions was outside the scope of this work, but would be warranted based on the large discrepancy between the NHD and our estimates of potential stream density.

Beside regulation and permitting of land use activities, there are many scientific applications for the stream maps generated here. For the first time we have an accurate representation of where streams once flowed through major urban areas of Baltimore and Washington, DC. These data are critical for quantifying the impact of urbanization on aquatic ecosystems and provide a means to quantify the loss and fragmentation of aquatic habitats on par with what has been available for terrestrial habitats for decades. Analyses of stream biogeochemistry and habitat loss should use updated stream maps and the NHD should be updated to reflect new information on stream presence or absence. Models attempting to accumulate landscape loading of nutrients to streams or otherwise simulate the hydrologic transport of materials through watersheds would benefit from using these high-resolution stream maps. The approach presented here is applicable wherever digital elevation models are available and suitable training data can be acquired. Improved stream maps, iteratively updated through field surveys and improved digital elevation models, are critical to forming sustainable development plans and understanding the functioning of Earth’s hydrologic system.
